# The management of hyperglycaemia in patients on enteral nutrition in Beaumont Hospital

**DOI:** 10.1186/1753-6561-6-S4-P41

**Published:** 2012-07-09

**Authors:** R Tully, H Giuden, E Fanning

**Affiliations:** 1 Royal College of Surgeons in Ireland, Ireland

## Objectives

To audit current practices in the management of hyperglycaemia in in-patients receiving enteral nutrition in Beaumont Hospital. The aims include:

• To examine the use of specific enteral feeds and their effects on hyperglycaemia

• To examine the medical management of hyperglycaemia

## Methods

Setting: A quantitative retrospective audit was carried out over 4 months. The medical charts and dietetic record cards of patients with raised blood glucose levels (defined as ≥8mmol/L) who were receiving enteral nutrition in Beaumont Hospital were collected by the department of Nutrition and Dietetics during the months of May to August.

Patients: Medical charts and dietetic record cards of patients with raised blood glucose (BGLs) levels whilst receiving enteral nutrition during the months of May to August 2011 were included in this audit. Twenty-four patient’s charts were eligible for the audit.

Main-outcomes/ measures: Use of diabetes specific feeds versus standard feeds, mean BGL (mmol/L) on each feed.

## Results

Of the 24 patients, 21% percent (n=5) of patients were started on a diabetes specific feed as a first line. 25% (n=6) of patients were changed to a diabetes specific feed during the audit. 66%percent (n=16) of patients were on insulin. 42% (n=10) of patients had endocrine team involvement. It was found that 46% (n=11) of patients were on a diabetic specific feed during their admission. Furthermore there was only a slight reduction in average high BGLs for these patients when compared to patients not on diabetic specific feeds.

## Conclusions

The aims of this audit were met by examining the use of specific enteral feeds and their effects on hyperglycaemia.

